# Factors affecting prefabricated construction promotion in China: A structural equation modeling approach

**DOI:** 10.1371/journal.pone.0227787

**Published:** 2020-01-27

**Authors:** Wen Jiang, Zhu Huang, Ying Peng, Yaqi Fang, Yunzhong Cao

**Affiliations:** College of Architecture and Urban-Rural Planning, Sichuan Agricultural University, Chengdu, Sichuan, China; Univerza v Mariboru, SLOVENIA

## Abstract

Prefabricated construction (PC) has attracted wide spread attention as a model of sustainable development for the construction industry of the future. Although the PC has many advantages, it is still at an initial stage in China. Based on the current conditions in China, this study focuses on the interrelationships of factors affecting PC promotion. Firstly, through a comprehensive review of relevant literatures and expert recommendations, 5 factors were identified: policy factor, technical factor, management factor, market factor and cost factor. Next, the data were collected through a questionnaire survey, and the questionnaire data were processed using SPSS 24.0 and AMOS 22.0. The overall relationships of each factor were quantitatively analyzed with Structural Equation Modeling (SEM). The results show that the policy factor plays a dominant role, while the management factor and market factors are also significant. This study also provides decision makers with relevant information about the factors involved, which will be helpful in devising appropriate strategies for the wider adoption of PC.

## Introduction

With the economic growth and urbanization, Chinese urbanization process will reach 60% by 2020 [1,2]. The "National New Town Planning" issued by the government indicates that 30 billion square meters of buildings will be built by 2020. Accordingly, the Chinese construction industry faces problems such as high waste generation rate, high risk and large labor demand. The application of prefabricated construction (PC) could solve such problems to meet the huge demand for housing. To promote sustainable development, Chinese governments have issued a series of policy documents to improve PC promotion. In 2016, the Central Committee of the Communist Party of China and the State Council issued "Several Opinions on Further Strengthening the Management of Urban Planning and Construction", demonstrating that it will take about ten years to increase the proportion of PC to 30% of new buildings. Then in 2017, the Ministry of Housing and Urban-Rural Development issued "13th Five-Year Planned Construction Action Plan", which set development goals for 2020 and clearly proposed key goals for the future.

PC is a manufacturing process, generally taking place at a specialized facility where materials are joined to form component parts of the final installation [3]. The advantages of PC have been studied extensively. These advantages include reduced construction waste, optimized quality management, reduced noise and dust, higher health and safety standards, reduced labor requirements, time and cost saving, accelerated project construction and reduced maintenance cost when compared with conventional construction techniques.

After the Second World War, European countries began to develop prefabricated components such as prefabricated beam plates and columns to meet the huge demand for housing reconstruction [4]. To promote the use of PC technology, the Danish government established prefabricated component standards, resulting in an increase of PC to 40% of total construction [5]. Hong Kong introduced PC in the mid-1980s and the Housing Authority recommended that public housing projects use prefabricated components in combination with standard modularity [6]. By 2002, prefabricated components accounted for 17% of the total concrete in public housing projects [7]. Driven by the Housing Authority, PC became very popular in public projects. In 2005, the proportion of assembly reached 65% in a pilot project [8].

The popularity of PC in China is low, and PC is still in its infancy. Due to the high costs and lack of standard specifications in the industry, most companies still use traditional construction methods. Compared to other countries, China has problems such as weak scale of economy and a lack of skilled workers, which leads to higher cost of prefabrication technology. With the acceleration of urbanization in China and increasing demand for housing, prefabricated buildings can provide an innovative solution to relieve housing pressure.

Through the analysis of literature review and actual conditions, this study summarizes five factors affecting PC promotion, including policy, technology, management, market and cost factors. This study uses exploratory factor analysis and confirmatory factor analysis to establish SEM for empirical analysis. Through data analysis, it shows that policy factor has the greatest impact on the PC promotion and other factors interact with each other to affect the PC promotion. In time, the results can help the government and administration to find workable solutions.

## Literature review

Some experts and scholars at home and abroad have explored and analyzed PC promotion. Arif M et al. [9] indicated that the degree of policy perception by construction enterprises strongly correlates with the promotion effect of PC. Enterprises are willing to accept policy guidance for transforming and upgrading, dependent on: perceived benefits from PC and the government's participation in improving sustainable development of the construction industry. At the same time, the views of participants in the industry may impact the application of PC technology [10]. It is believed that the slow development of PC is due to its own high cost and complicated application of technology. Aside from high construction cost, there are difficulties in on-site construction and installation management of fabricated concrete structures. Steinhardt D.A et al. [11] analyzed the use of prefabricated components in the housing industries of Australia, Japan, Sweden, Germany and other countries. It was found that under certain conditions, market demands, public preferences, and national policy support can drive the use of prefabricated components. The cost-effectiveness of prefabricated components in China is a key issue for all stakeholders. Therefore, the government should implement policies to remove the economic barriers, encourage stakeholders to adopt PC, and train workers by offering appropriate technical guidance, effectively reducing the upfront cost [12]. Foxon et al. [13] stressed that policy factor will affect the technological innovation of PC. The government should vigorously cultivate professional talents, fill gaps in industry standards, increase research input and encourage innovation. A construction enterprise’s satisfaction with PC is mainly affected by time, cost and productivity. However, construction companies are unwilling to adopt PC techniques because of high capital costs, difficulties in achieving economies of scale, immature production of prefabricated components, enterprise culture of avoiding risks[14,15], and lack of experience in operating such projects [16]. The adoption of a conservative strategy [17] established a dynamic assembly simulation dynamic model, analyzing the factors affecting assembly progress from three levels, including inefficient quality inspection and design data conversion, asynchronous data in the industry chain, and poor collaboration among enterprises due to design changes. It is believed that the application of building information modeling (BIM) technology will help to establish a standardized design system, improve the efficiency of contractors in the design and construction phase, and reduce the labor time [18].

Foreign scholars have conducted extensive researches on PC promotion, which is the foundation for this study. However, most foreign studies, such as in Australia and the United States, are based on their own national conditions or overall world conditions, and mostly focus on one aspect of PC promotion. Most studies in China mostly adopt qualitative methods, but lack researches on the mechanism of influence among various factors. Based on actual conditions in China, this study uses SEM to explore the relationships between various factors affecting PC promotion, and conducts empirical tests to provide reference values for PC promotion strategy in China.

## Methodology

Structural equation modeling (SEM) is an advanced statistical method developed from factor analysis and path analysis. Fisher R.A et al. [19] proposed path analysis in genetics and introduced the path map to obtain the basic form of the SEM in 1918. Joreskog K.G et al. [20] put forth the preliminary concept of SEM in the early 1970s, dividing the SEM into structural models and measurement models. Since then SEM has been widely used in biology, medicine, education, behavior, psychology and many other fields.

In the field of construction, Saheed O.A et al. [21] quantitatively analyzed a set of measures using SEM, and confirmed their effectiveness in reducing construction waste in the building demolition process. Mohammad Zaira M et al. [22] used SEM to analyze the relationship between safety interventions and practices on a construction site, so as to improve systematic and comprehensive safety management in construction. Yang W et al. [23] used SEM to evaluate the social impact of construction projects and their relationship to public response to identify and solve conflicts from a social risk management perspective.

### Research methods

SEM is a statistical technique that can simultaneously measure and analyze. It can estimate potential variables and error variables that are difficult to observe in the model directly. Through SEM, the causal relationships between the model and the verification model can be identified and verified. SEM is divided into two parts: the measurement model and the structural model. The measurement model describes the relationships between the latent variables and the observed variables (1, 2), and the structural model describes the relationships among the latent variables (3). SEM can be constructed and analyzed with SPSS24.0 and AMOS22.0 software.

Measuring model equations are [24]:

X=ΛXξ+δ (1)

Y=ΛYη+ε (2)

Structural model equation is [24]:

η=Γξ+ζ (3)

*ΛX* and *ΛY a*re the factor loadings of the index variables (*X,Y*), δ and ε are the measurement errors of the explicit variables, *ξ* and *η* are the exogenous latent variables and endogenous latent variables respectively. *ζ* is the residual, Γ is the matrix of structural coefficient between the latent variables and the external dependent latent variables.

SEM is aimed at normal distribution data and it is very important to select appropriate estimation methods. Maximum likelihood (ML) technique is relatively powerful for data in normal state, so this study uses ML technique to estimate model variables.

### Research hypothesis

**Fig 1:**
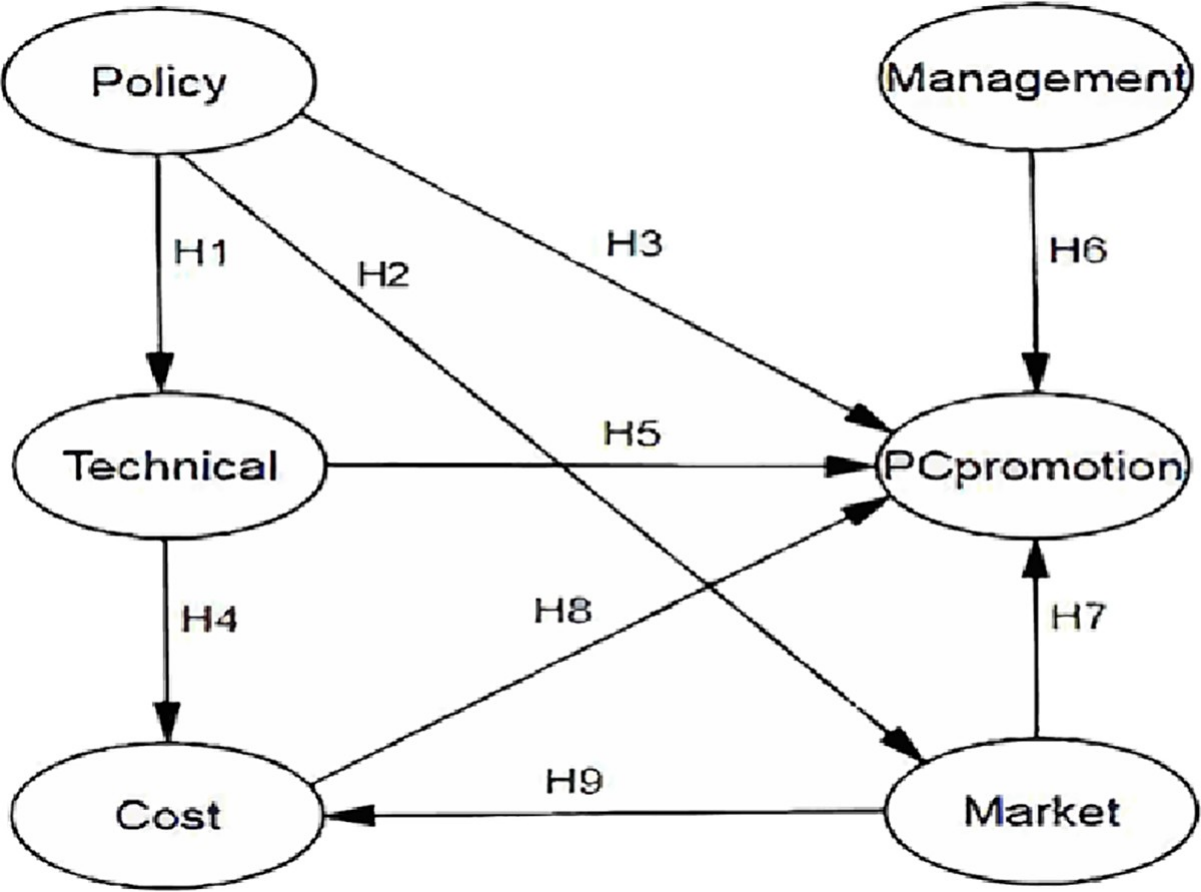
Conceptual framework model.

When using SEM for analysis, it is necessary to assume the path relationships of the model concept map in order to measure the relationships among the variables. The conceptual framework diagram is shown in Fig 1. Based on literature review, the relationships among the theoretically analyzed variables and the assumptions of each path are shown in Table 1.

**Table 1:** Research hypothesis.

Item	Path hypothesis			Impact
H1	Policy factor	→	Technical factor	Significant impact
H2	Policy factor	→	Market factor	Significant impact
H3	Policy factor	→	PC promotion	Significant impact
H4	Technical factor	→	Cost factor	Significant impact
H5	Technical factor	→	PC promotion	Significant impact
H6	Management factor	→	PC promotion	Significant impact
H7	Market factor	→	Cost factor	Significant impact
H8	Market factor	→	PC promotion	Significant impact
H9	Cost factor	→	PC promotion	Significant impact

### Factor identification

Through a comprehensive review of PC literatures, this study identified the provisional list of factors shown in S1 File. Semi-structured interviews with experts were conducted to confirm the validity and reliability of factors. These experts recommended by university professors have more than 10 years of construction experience. Otherwise, they are from university and construction enterprises. The detailed information about experts and interview arrangements is saved in S2 File. The phases of interviews are as follows:

1. We got verbal consent from experts by telephone and reached agreements about interview time.
2. We sent the provisional list to experts via email and received confirmation feedback.
3. We conducted interviews with experts from November 2018 to December 2018 and every interview lasted between 45 and 60 minutes.

During the interviews, we read out the consent letter which is saved in S3 File and the experts agreed to conduct the next interview. Then, every expert was asked to answer the following questions:

1. What do you think should be emphasized in the design of the questionnaire?
2. Do you think the provisional list of factors is appropriate and which factor needs to be deleted or added?
3. Can you talk about your views on Chinese prefabricated construction industry?
4. What do you think should be paid attention to in the selection of the survey object in this study?

Given Chinese PC industry background, experts gave reasonable advice on provisional list. Finally, 18 observed variables were identified. The study divided the 18 observed variables into policy factor, technical factor, management factor, market factor, cost factor, and PC promotion, as shown in Table 2.

**Table 2:** Sources of observed variables.

Potential variables	Observation variables	Literatures	Item
PF	Policy incentive	[13]	X_11_
	Industry standard	[45],[40],[33]	X_12_
	Regulatory mechanism	[8],[7],[42]	X_13_
TF	BIM technology	[17],[29],[32]	X_21_
	Standardization	[40],[33],[43]	X_22_
	Technical talent	[17],[7],[12],[41]	X_23_
MF	Organizational strategy	[37]	X_31_
	Information collaboration	[44] [38]	X_32_
	Management mode	[38],[30],[39]	X_33_
MF	Industry chain	[7],[34],[31]	X_41_
	Public acceptance	[1],[35]	X_42_
	Enterprise transformation	[39],[1],[5],[24]	X_43_
CF	Production cost	[36]	X_51_
	Purchase cost	[43]	X_52_
	Transportation cost	[5],[37],[36]	X_53_
PCP	Environmental performance	[10]	X_61_
	Economic performance	[10]	X_62_
	Social performance	[10]	X_63_

[1] Gan X.L et al. (2018); [5] Jaillon L et al. (2009); [7] Chiang, Y.H et al. (2006); [8] Tam V.W.Y et al. (2007); [10] Li Z.D et al. (2014); [12] Hong J.K et al. (2018); [13] Foxon T.J et al. (2007); [17] Li C.Z et al. (2018); [29] Abanda, F.H et al. (2017); [30] Blismas N et al. (2009); [31] Dewick P et al. (2004); [32] Eastman, C.M et al. (2008); [33] Gan Y et al. (2017); [34] Hamid Z.A et al. (2011); [35] Jaillon L et al. (2010); [36] Li C.Z et al. (2016); [37] Lu W et al. (2013) [38] Luo L.Z et al. (2015); [39] Mao C et al. (2015); [40] Polat G et al. (2008); [41] Polat G et al.(2010); [42] Teng, Y et al. (2017); [43] Wang J et al. (2016); [44] Xue X et al. (2018); [45] Zhang, X.L et al. (2014).

#### Policy factor

*Policy incentive*. The government incentives for the implementation of PC are insufficient. There is no support for policies such as land transfer and construction bidding policies. The government only provides policy allowance to property owners, and has not issued effective policies for the design and production participants.

*Industry standard*. Due to a lack of global uniform standards in the world, prefabricated components cannot be produced efficiently. In addition, Chinese PC lacks production specifications and quality testing standards, thus mechanical properties such as strength and stability of components cannot be guaranteed.

*Regulatory mechanism*. A perfect regulatory mechanism can highlight the advantages of PC, effectively reduce construction costs and ensure accuracy in assembly. Most local governments have not developed a matching regulatory system, and the Chinese regulatory mechanism is immature.

#### Technical factor

*BIM technology*. In the whole life cycle, BIM technology can accurately reflect the size, structure and performance of each component in digital form. In addition, BIM can formulate effective procurement strategies, reduce waste of building materials on site, and promote the assembly of standard components. However, those who master BIM technology in China are few, and the level of application is low, resulting in construction rework at the construction site, which hinders the project management schedule.

*Standardization*: There is no adaptive modular system in Chinese PC production, resulting in component incompatibility. Thus, the successful connection of prefabricated components is hindered, which complicates design and production and results in a lower output.

*Technical talent*. There are no experienced suppliers, contractors and designers in the PC market. In a highly competitive market, experienced suppliers will provide high-quality components at reasonable prices, designers will deliver diverse designs, and contractors will be more familiar with PC methods. The lack of technical personnel may lead to building components a series of problems such as poor structural performance and inferior site management.

#### Management factor

*Organizational strategy*. Organizational strategy will greatly affect the PC promotion. The quality of the project management depends on the understanding of project members and their sense of responsibility in the whole process.

*Information collaboration*. If the participants in a PC project lack coordination, reliable transmission of information and timely resolution of problems are not possible. Fragmentation of information in the construction industry is one of the factors hindering PC promotion. The industry has not integrated the process of design, production, transportation, assembly and maintenance into an information platform, which may cause mismatched structural design, lack of qualified labor, low-quality production of prefabricated components, and higher transportation cost.

*Management mode*. PC promotion requires multi-participation management and deep cooperation among partners. The new construction management system should be adopted in bidding, quality supervision and completion acceptance. Traditional on-site schedule management is not suitable for PC, so participants need to plan well in advance.

#### Market factor

*Industry chain*. The market factor plays a decisive role to in improving the industrial chain in a mature industrialization model. The lack of innovation and application of PC technology is largely due to a lack of cooperation among stakeholders. They cannot effectively coordinate all elements in the whole life cycle of PC products, resulting in the fragmentation and discontinuity of the industrial chain.

*Public acceptance*. The public acceptance as an external environment plays a vital role in PC promotion. Market demand is limited by a negative perception and the lack of understandings of PC.

*Enterprise transformation*. Leading companies have not championed the use of prefabricated components. Further, there is no systematic and comprehensive understanding of the industry chain within the industry. Private companies still depend on traditional on-site construction processes but the PC promotion requires companies to invest heavily in equipment upgrades, talent training and prefabrication technology.

#### Cost factor

*Production cost*. The purchase of new machines, production of molds and the development of prefabricated parts technology will make it difficult to raise funds in the early stage of PC production. Such investment will result in a longer payback period and higher amortization cost for components. The current technology of prefabricated components in China is immature and the scale of production is small, which leads to higher production cost.

*Purchase cost*. A component is transitions from the on-site construction to the prefabricated assembly in the factory. The 17% value added tax (VAT) rate is significantly higher than the tax rate in the civil construction sector, which will increase the cost of purchase and hinder PC promotion.

*Transportation cost*. Transportation cost accounts for 6%-11% of the total cost. The Manufacturers build factories in remote areas in order to reduce cost, but increases the mileage in the transportation process. To avoid damage, the suppliers need to strictly control and properly fix the prefabricated components.

#### PC promotion

*Environmental performance*. PC can reduce the amount of construction waste generated, improve the recycling rate of resources and promote sustainable development during all stages of the life cycle.

*Economic performance*. PC can reduce the cost throughout the life cycle, increase the income of construction enterprises and improve management of the industrial chain.

*Social performance*. PC can drive industrial productivity, create new jobs, promote industry development and improve the quality of public life.

### Data collection

Based on the conceptual model and the measurement index system, the relevant expert recommendations are combined and the questionnaire is finalized through multiple rounds of group discussions. The questionnaire measures the variables in the form of a Likert 5 scale. The snowball sample technique was used to deliver the questionnaire, which ensures wide and efficient delivery to similar people. The questionnaire was first delivered to the participants who were in long-term cooperation with college of university through the recommendation of senior professors. They have rich knowledge in the PC field and understand industry policy very well. In addition, the questionnaire was delivered via email, QQ, WeChat and online Respondents were also asked to forward the questionnaire to their colleagues or friends in the PC field. After their nomination, we conduct the snowballs sampling. The final sample survey was completed through several rounds of distributing questionnaires.

A total of 415 questionnaires were issued (online, or offline in paper form), to the real estate enterprises, design enterprises, construction enterprises, engineering consulting enterprises, materials and equipment enterprises, research institutions and government administration. The number of effective questionnaires was 371 (89.39% recovery rate) and questionnaire data is saved in S4 File. There is no any ethical oversight in this study and participants gave their consent when completing the questionnaire.

## Data analysis

### Exploratory factor analysis

The main purpose of exploratory factor analysis is to determine the number of observed variables. Before formal factor analysis, we conducted exploratory analysis on 371 valid samples. At this stage, the reliability and validity tests are to check data reliability and question consistency. The multivariate normality of the data is determined by describing the skewness and kurtosis of the observed variables. After satisfying the above requirements, the principal component analysis method is used for factor analysis and six comprehensive factors are extracted. After skew rotation, they fully reflect the factors of PC promotion. The results provide a basis for the establishment of hypotheses for confirmatory factor analysis.

### Reliability test

This study uses SPSS24.0 to obtain the Cronbach's alpha coefficient and analyze the reliability of the scale. It is observed from the results that the overall Cronbach's alpha coefficient is 0.926, and the Cronbach's alpha coefficient values of the six latent variables are all greater than 0.6, indicating that the overall reliability of the questionnaire data is good. The value of the Cronbach's alpha coefficient is shown in Table 3.

**Table 3:** Reliability test results for each dimension.

Latent variables	measurable variables	Cronbach's alpha coefficient
PF	X_11_	0.875
	X_12_	
	X_13_	
TF	X_21_	0.881
	X_22_	
	X_23_	
MF	X_31_	0.856
	X_32_	
	X_33_	
MF	X_41_	0.875
	X_42_	
	X_43_	
CF	X_51_	0.866
	X_52_	
	X_53_	
PCP	X_61_	0.891
	X_62_	
	X_63_	

#### Validity test

Bartlett's sphere and KMO tests were performed using SPSS 24.0. The results show that the overall KMO value of the questionnaire is 0.899 and the KMO values of the six latent variables are greater than 0.5. The Sig value of the Bartlett sphere test is 0, which is less than 0.01, indicating that the sample data is highly correlated. The above results show that the sample data have good convergence validity.

Skewness and kurtosis can effectively describe the normal distribution of data for each observed variable. The values of skewness and kurtosis are shown in Table 4 within ±2 [25].The average value of skewness and kurtosis of each observed variable is 0.760–0.406. All variables and averages are in accordance with the standard, which indicates the data show a normal distribution.

**Table 4:** The values of skewness and kurtosis.

	X11	X12	X13	X21	X22	X23	X31	X32	X33	X41	X42	X43	X51	X52	X53	X61	X62	X63
Skewness	-0.687	-0.749	-0.829	-0.744	-0.844	-0.953	-0.856	-0.666	-0.732	-0.808	-0.782	-0.810	-0.681	-0.538	-0.756	-0.743	-0.746	-0.749
Kurtosis	-0.456	-0.281	-0.387	-0.370	0.073	0.134	-0.021	-0.247	-0.468	-0.047	-0.152	0.088	-0.306	-0.706	-0.233	-0.105	-0.381	-0.406

#### Factor analysis

Factor analysis is used to characterize the basic structure of data by analyzing the internal mapping relationships among variables. In this study, Principal Component Analysis (PCA) is used to obtain the results using SPSS 24.0. The basic function of factor analysis is to analyze the mapping relationships within the data matrix. Factor analysis can group variables according to the mapping degree, extract the key indices of different groups and calculate the cumulative contribution rate of variance. These key indices can directly reflect the basic structure of things. Table 5 shows that the exploratory factor loading of 18 indicators is obtained by the maximum variance rotation analysis. The quantities are all greater than 0.5, which meets the model requirements and allows extraction of six factors to explain the structure of the variables. After the skew rotation, the latent variables explain 80.419% of the initial information, fully reflecting the factors of PC promotion.

**Table 5:** Results of PCA and rotated factor analysis.

	1	2	3	4	5	6
**Policy incentive**	.819					
**Industry standard**	.787					
**Regulatory mechanism**	.817					
**BIM technology**		.831				
**Standardization**		.840				
**Technical talent**		.832				
**Organizational strategy**					.828	
**Information collaboration**					.789	
**Management mode**					.822	
**Industry chain**				.818		
**Public acceptance**				.816		
**Enterprise transformation**				.840		
**Production cost**			.820			
**Purchase cost**			.805			
**Transportation cost**			.835			
**Environmental performance**						.772
**Economic performance**						.781
**Social performance**						.819
**Eigenvalues**	7.980	1.491	1.383	1.362	1.218	1.041
**Variance interpretation %**	44.336	8.282	7.684	7.568	6.766	5.785
**Cumulative variance interpretation %**	44.336	52.618	60.301	67.869	74.635	80.419

### Confirmatory factor analysis

**Fig 2:**
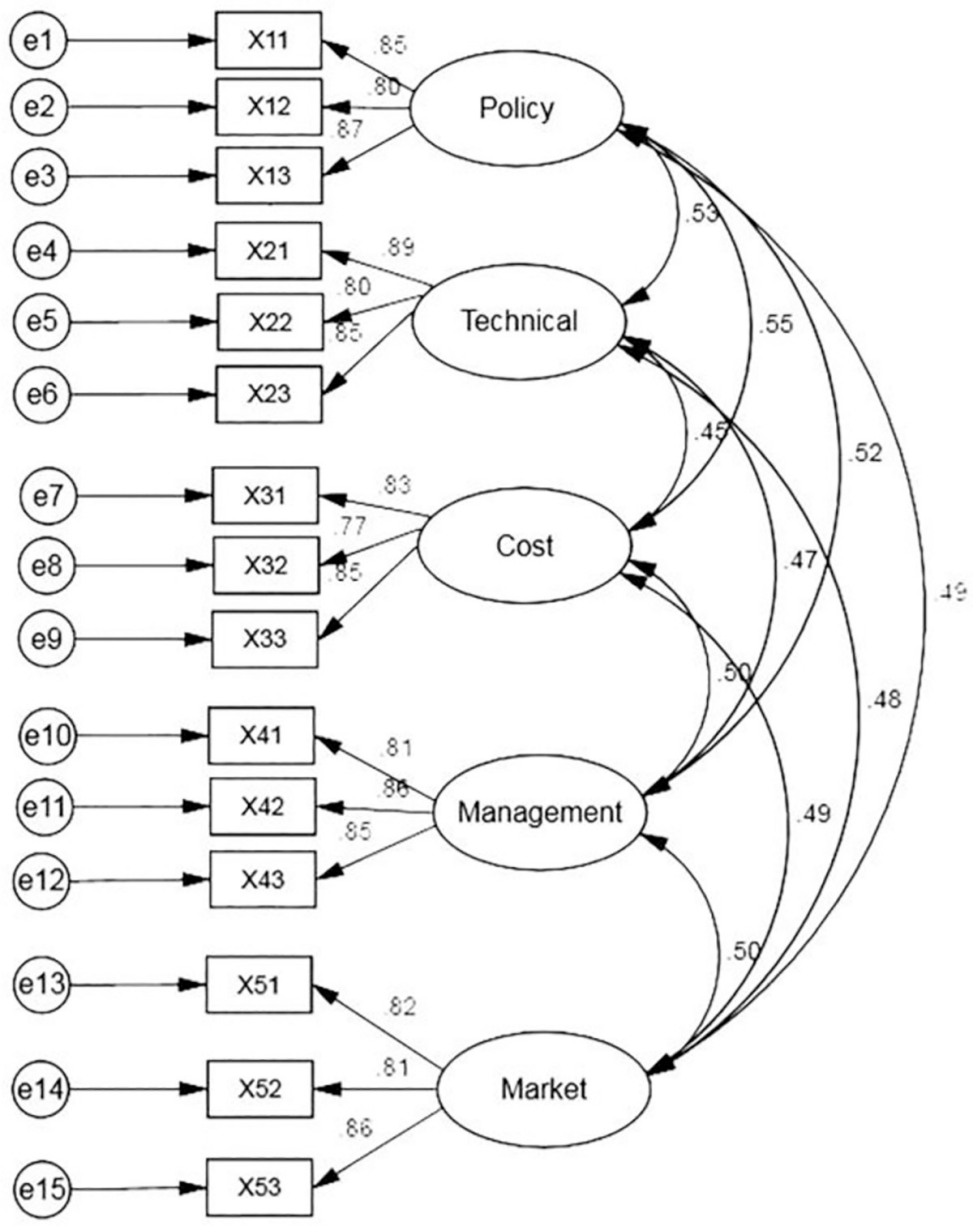
Initial model of PC promotion.

CFA is conducted to confirm the quality and adequacy of the measurement model. When using normally distributed data, it is suitable to use ML for model estimation [26]. The correlation coefficient matrix is shown in Table 6. All observed variables correlation coefficient values are from 0.229 to 0.759, which indicates significant correlation. The initial model is established as shown in Fig 2. Before evaluating the model fitness, it is necessary to first test the "Offending Estimates" to determine whether the estimated coefficient is within the acceptable range. Hair et al. [27] believes that the offending estimate follows three rules. First, there is a negative error term variation. Second, the standardization coefficient is too close to or exceeds 1 (generally 0.95 is the threshold), and third, the standard error is too large. If the test results don’t have the aforementioned offending estimation characteristics, the preliminary test of the model is qualified, and the fitness test can be carried out.

**Table 6:** Correlation coefficient matrix.

	X11	X12	X13	X21	X22	X23	X31	X32	X33	X41	X42	X43	X51	X52	X53	X61	X62	X63
X11	1																	
X12	0.669**	1																
X13	0.745**	0.694**	1															
X21	0.417**	0.402**	0.375**	1														
X22	0.319**	0.371**	0.346**	0.713**	1													
X23	0.433**	0.360**	0.376**	0.747**	0.679**	1												
X31	0.376**	0.378**	0.400**	0.344**	0.366**	0.329**	1											
X32	0.420**	0.332**	0.365**	0.305**	0.229**	0.286**	0.632**	1										
X33	0.362**	0.370**	0.402**	0.339**	0.251**	0.302**	0.715**	0.655**	1									
X41	0.400**	0.339**	0.351**	0.321**	0.300**	0.368**	0.324**	0.400**	0.349**	1								
X42	0.377**	0.364**	0.381**	0.352**	0.307**	0.364**	0.328**	0.318**	0.368**	0.686**	1							
X43	0.347**	0.371**	0.366**	0.318**	0.316**	0.350**	0.335**	0.330**	0.344**	0.685**	0.728**	1						
X51	0.379**	0.375**	0.364**	0.373**	0.343**	0.321**	0.338**	0.335**	0.346**	0.320**	0.358**	0.268**	1					
X52	0.328**	0.337**	0.359**	0.362**	0.341**	0.326**	0.322**	0.350**	0.337**	0.323**	0.359**	0.343**	0.659**	1				
X53	0.340**	0.326**	0.322**	0.363**	0.322**	0.314**	0.345**	0.337**	0.340**	0.388**	0.367**	0.366**	0.709**	0.694**	1			
X61	0.454**	0.484**	0.502**	0.421**	0.381**	0.381**	0.358**	0.398*	0.447**	0.391**	0.455**	0.446**	0.390**	0.421**	0.424**	1		
X62	0.386**	0.419**	0.447**	0.446**	0.405**	0.401**	0.377**	0.428**	0.424**	0.368**	0.414**	0.379**	0.432**	0.431**	0.697**	0.697**	1	
X63	0.450**	0.446**	0.493**	0.416**	0.350**	0.347**	0.339**	0.407**	0.441**	0.368**	0.457**	0.401**	0.438**	0.427**	0.759**	0.759**	0.745**	1

** correlation is significant at the 0.01 level (2-tailed).

To evaluate the internal structure fit, average variance extracted (AVE) can be used to assess the significance of the estimated parameters in the model, the indices and the reliability of the latent variables. In addition, composite reliability (CR) is used to evaluate the consistency of the measured variables. The CR should be greater than or equal to 0.6 and the AVE test should be greater than 0.5 to meet the intrinsic quality verification analysis standard of the model. Table 7 indicates the internal structure of the model has good fitness. Table 8 shows the fit index calculations, where χ^2^/df is 1.467 (<3), RMSEA is 0.036 (<0.08), GFI is 0.959 (>0.9), NFI is 0.966 (>0.9), IFI is 0.989 (>0.9), CFI is 0.989 (>0.9), TLI is 0.985 (>0.9), PGFI is 0.640 (>0.5), PNFI is 0.736 (>0.5) and PCFI is 0.753 (>0.5). The measurement model appears to be acceptable, as the above indices are in line with the evaluation criteria, indicating the model-fit is good.

**Table 7:** Intrinsic quality verification factor analysis.

Potential variables	Construction reliability	Average variance extraction	Criteria	Discriminant result
Policy factor	0.8777	0.7055		well
Technical factor	0.8821	0.7140	Construction reliability ≥0.6	well
Management factor	0.8754	0.7010		well
Market factor	0.8687	0.6882	Average variance extraction >0.5	well
Cost factor	0.8568	0.6664		well

**Table 8:** Fit index calculation.

Indices	CFA	SEM	Suggested value
χ^2^/df	1.467	2.93	<3
RMSEA	0.036	0.072	<0.08
GFI	0.959	0.904	>0.9
NFI	0.966	0.936	>0.9
IFI	0.989	0.964	>0.9
CFI	0.989	0.964	>0.9
TLI	0.985	0.964	>0.9
PGFI	0.64	0.666	>0.5
PNFI	0.736	0.754	>0.5
PCFI	0.753	0.776	>0.5

### Test of the SEM constructs and correlations

**Fig 3:**
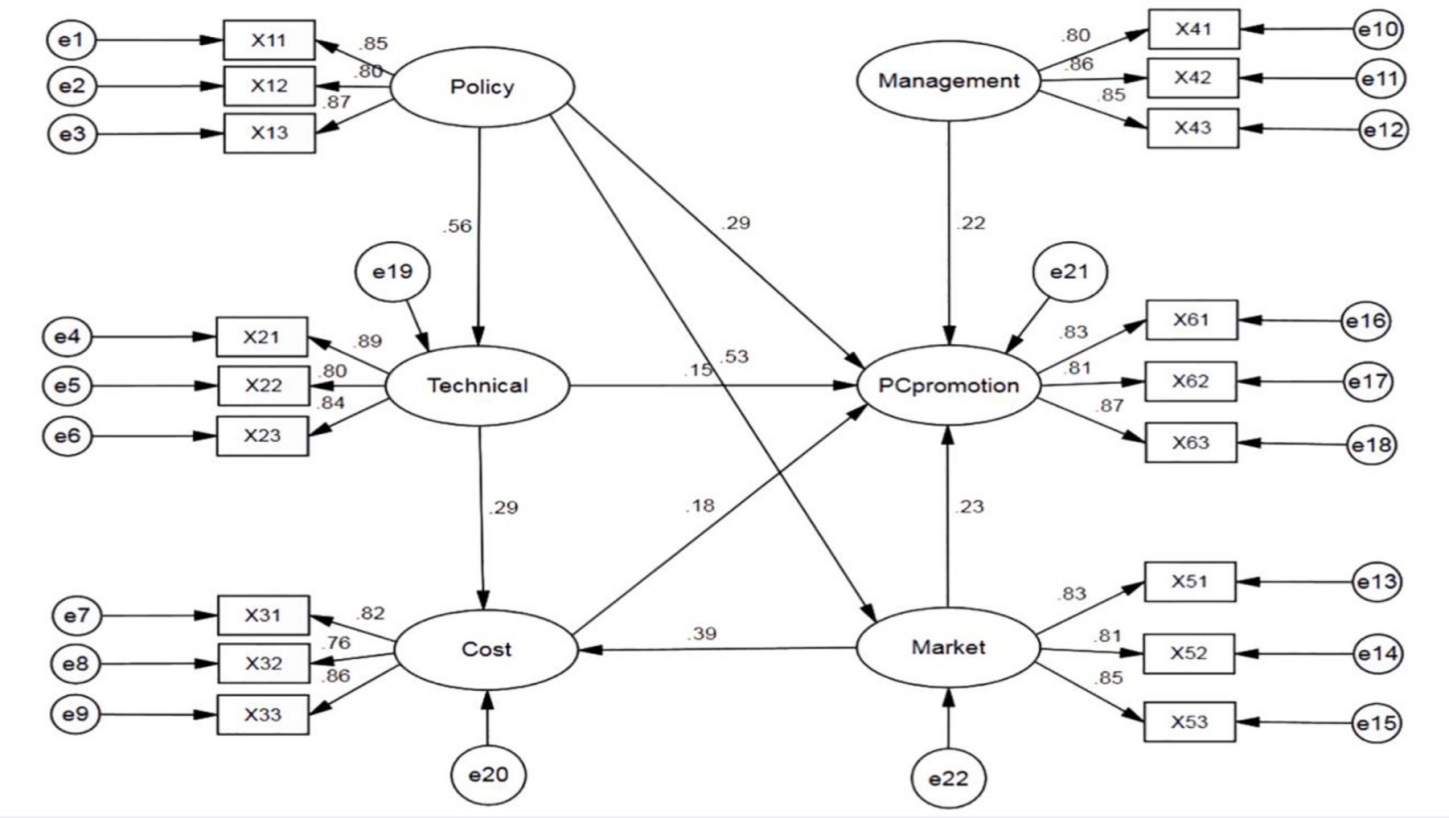
Structural equation modeling diagram of factors of PC.

SEM can replace the correlations among constructs with the proposed causal relations in the theoretical model and refine the models further [28]. A new SEM is developed to examine the relationships between the five influencing factors to determine how to promote the PC in the future. From Fig 3, the relationships between the independent and dependent variables can be measured by a path diagram. As shown in Table 8, the same indices can be used to evaluate adequacy of both the CFA and SEM. All indices are in the appropriate range, indicating the model is acceptable. All hypotheses are supported by the data in Table 9. The p values between the potential variables are all less than 0.05, so it is assumed that H1, H2, H3, H4, H5, H6, H7, H8 and H9 are all established. The regression coefficients of cost and technology are small. However, the p value meets the requirements, thus the conclusion is reliable. Five factors are positively correlated with PC promotion.

**Table 9:** Model path coefficient fitting.

Path hypothesis			Standardized estimate	S.E.	C.R.	P	Test result
Policy factor	→	Technical factor	0.556	0.048	9.913	***	Significant impact
Policy factor	→	Market factor	0.528	0.046	9.004	***	Significant impact
Policy factor	→	PC promotion	0.291	0.051	4.297	***	Significant impact
Technical factor	→	Cost factor	0.293	0.065	4.836	***	Significant impact
Technical factor	→	PC promotion	0.151	0.053	2.471	0.013	Significant impact
Management factor	→	PC promotion	0.219	0.054	3.819	***	Significant impact
Market factor	→	Cost factor	0.387	0.075	6.162	***	Significant impact
Market factor	→	PC promotion	0.233	0.061	3.704	***	Significant impact
Cost factor	→	PC promotion	0.184	0.050	3.011	0.003	Significant impact

*** p < 0.001

## Discussions and suggestions

### Discussions

The policy factor has a path coefficient of 0.291 for PC promotion, which has the biggest impact on PC promotion. There is no uniform specification for PC in China and industry standard norms are the cornerstones of PC. Unlike traditional perceptions, it is not industry standards but regulatory mechanisms that have the greatest impact on PC promotion in the policy system. The establishment of a supervision mechanism can enable contractors to plan, execute, inspect and rectify deviations in accordance with engineering standards. Thus, a supervision mechanism can ensure the quality, safety and construction progress of engineering construction. In addition, the policy factor can indirectly affect PC promotion through technology and market, with the belief that government policies are beneficial to promote technological innovation, increase public acceptance, and accelerate PC promotion.

The cost factor has a path coefficient of 0.184 for PC. Because PC is in the initial stage in China, the costs will be higher, which will hinder the PC promotion to some extent. However, from the perspective of development prospects, when the technical standards, policy indicators and market environment are mature, the costs will decrease. Thus, the cost factor is mainly affected by the interaction of other factors, which is why it has a low impact on PC promotion. The cost of prefabricated components (from production, to transportation, to assembly) is higher than that of traditional construction. The establishment of prefabricated factories requires new machines and equipment during the transportation process, and more manpower and material resources to carry out maintenance on the components. Prefabricated components are products that highly commercialize building materials, increasing VAT from 10% to 17%. The price of prefabricated components will increase accordingly.

The market factor path coefficient for PC promotion is 0.233. Limited market demand often prevents the promotion of new things, and PC feasibility is still considered suspect by the public and the construction companies. Subject to the influence of traditional and conservative culture, the public is skeptical about PC, which leads to lower market demand. It is difficult for construction enterprises to break from the tradition due to the uncertainty and market risks. Thus, there is reluctance to transform and upgrade. The market factor indirectly affects PC promotion through cost factor. Due to the conservation of enterprises, the fragmented market chain hinders communication between upstream and downstream industries, raising the cost.

The technical factor has a path coefficient of 0.151 for PC promotion, showing that technology affects PC promotion but is not the main factor. Technology is constantly updated and improved. At this stage, the Chinese product system has been initially established and some of the technology and product quality has reached a more advanced level. However, the PC industry does not have a complete modular system, which results in a low degree of standardization. The current talent training mechanism is mainly aimed at traditional construction, and cannot develop new technical talents in the PC industry. Furthermore, talents in building information technology are scarce. The application of BIM technology in PC is low, which affects PC promotion.

The management factor path coefficient for PC promotion is 0.219. PC promotion requires companies to change their management model and adjust their corporate strategy. Each enterprise pursues its own interests and the high degree of independence and fragmentation in the construction industry makes information exchange and coordination difficult.

### Suggestions

PC promotion needs interaction between all parties involved. The issues need to be discussed before measures can be taken to promote PC. The development of the PC industry can start from the following points:

#### Pay attention to the formulation of policies and regulations

When introducing new policies, governments should integrate the interests of stakeholders in the industrial chain and fully consider the perspective of designers, manufacturers and contractors. The government should clarify the industry standard system and support construction enterprises. It is essential to establish an industry base to break through its technical barriers and control the production cost of PC. An informational supervision system should be established for oversight of the entire industry chain.

#### Focus on resource restructuring and industrial integration

Construction enterprises should actively respond to national policy calls to promote the integration in design, production and assembly. Construction enterprises can promote the transformation and upgrade of traditional on-site building, driving the industrial chain to improve. To enhance public acceptance, the government should emphasize the superior quality and performance of PC to the public, so that the market can promote new applications.

#### Pay attention to the application of new technologies

Scientific research institutions should adopt standardized design, which is conducive to factory integration, modular production and efficient assembly on the construction site. Universities and construction enterprises should focus on cultivating technical talents to fill current talent vacancies. Enterprises should apply BIM to the whole life cycle of the PC, including work schedule sharing and reduction in waste of resources in the assembly process.

## Conclusions

PC is still in the initial stage in China but it will undoubtedly be the direction of future Chinese building industrialization. This study ranks 18 factors based on relative research and uses SEM to identify empirical research. The results indicate that government plays a leading role in promoting PC. Among the policy factors, the most influential factor is not the industry standard but the regulatory mechanism. The conclusions of the study suggest the need for the improvement of the theoretical research system of PC.

There are limitations to this research. It should be noted that only 18 factors were chosen to demonstrate the state of PC promotion. Thus, the study is relatively imperfect. The regression coefficient of cost and technology may be smaller in the model than in actual conditions. In addition, the data collected in this study only reflect the current status of Chinese PC. Over time, the factors in the PC promotion process will change. As conditions in Chinese cities differ, further investigation can be based on specific cities. It is also feasible to conduct surveys in other developing countries, where PC may be an effective way to promote local industrial development.

## Appendix

### Research on factors affecting prefabricated construction promotion

Dear Gentleman/Madam

Thank you very much for taking time to fill out this questionnaire, which is an anonymous survey. This survey aims to study the factors affecting prefabricated construction promotion. All data are only used for academic research.

Your choice is crucial to our research! We sincerely thank you for your support and cooperation. If you agree to authorize, please fill in this questionnaire.

Research Group on Management Strategy and Coordination

Mechanism of Prefabricated Construction Supply Chain Driven by Big Data

January 4, 2019

Tips: The following questions are all single.

1. What is your **gender**?
  ○man             ○woman
2. What is your **age**?
  ○20~30 years old             ○31~40 years old
  ○41~50 years old             ○51 years old and above
3. What is your education **background**?
  ○junior college and below             ○undergraduate
  ○master                          ○doctor
4. Where is your **workplace**?
  ○real estate enterprise
  ○design enterprise
  ○construction enterprise
  ○engineering consulting enterprise
  ○material/equipment supply enterprise
  ○research institution
  ○government administration
5. How about the impact of **policy incentive** such as tax reduction and land transfer on the promotion of prefabricated construction?
  ○very small    ○small    ○general    ○big    ○very big
6. How about the impact of the **industry standard** (such as production standards and quality inspection standards) on the promotion of prefabricated construction?
  ○very small    ○small    ○general    ○big    ○very big
7. How about the impact of the **regulatory mechanism** on the promotion of prefabricated construction?
  ○very small    ○small    ○general    ○big    ○very big
8. How about the impact of **the application of building information modeling (BIM) technology** in prefabricated component design, production, installation, operation and maintenance on the promotion of prefabricated construction?
  ○very small    ○small    ○general    ○big    ○very big
9. How about the impact of **standardization** of modular system on the promotion of prefabricated construction?
  ○very small    ○small    ○general    ○big    ○very big
10. How about the impact of **prefabricated construction technical talents** on the promotion of prefabricated construction?
  ○very small    ○small    ○general    ○big    ○very big
11. How about the impact of **organizational strategy** (the overall goal of the company and the collaboration of individual employees) on the promotion of prefabricated construction?
  ○very small    ○small    ○general    ○big    ○very big
12. How about the impact of **information collaboration** (such as information delivery reliability, timely feedback and complete information channels) on the promotion of prefabricated construction?
  ○very small    ○small    ○general    ○big    ○very big
13. How about the impact of **management mode** (such as cooperation among all parties involved, project planning and innovative management tools) on the promotion of prefabricated construction?
  ○very small    ○small    ○general    ○big    ○very big
14. How about the impact of **industry chain** (the cooperation of stakeholders promotes the application of prefabricated technologies to form a complete industrial chain) on the promotion of prefabricated construction?
  ○very small    ○small    ○general    ○big    ○very big
15. How about the impact of **public acceptance** on the promotion of prefabricated construction?
  ○very small    ○small    ○general    ○big    ○very big
16. How about the impact of intention of **enterprise transformation** (enterprise changes the concept of traditional cast-in-place and adopts prefabricated buildings) on the promotion of prefabricated construction?
  - ○very small    ○small    ○general    ○big    ○very big
17. How about the impact of **production cost** (such as the cost of acquiring new machines, manufacturing molds and developing prefabricated technology in the production process) on the promotion of prefabricated construction?
  ○very small    ○small    ○general    ○big    ○very big
18. How about the impact of **purchase cost** (value-added tax for prefabricated components purchase is 17%, higher than the rate for other civil materials) on the promotion of prefabricated construction?
  ○very small    ○small    ○general    ○big    ○very big
19. How about the impact of **transportation cost** of components from factory to assembly site on the promotion of prefabricated construction?
  ○very small    ○small    ○general    ○big    ○very big
20. What is the degree of **environmental performance** (such as reducing the demand for construction waste and materials, increasing the recycling utilization rate of resources, promoting sustainable development) brought by promotion of prefabricated construction?
  ○very small    ○small    ○general    ○big    ○very big
21. What is the degree of **economic performance** (reducing whole life cycle cost, increasing income, improving industry chain management) brought by promotion of prefabricated construction?
  ○very small    ○small    ○general    ○big    ○very big
22. What is the degree of **social performance** (increasing productivity, creating new jobs, driving industry development) brought by promotion of prefabricated construction?
  ○very small    ○small    ○general    ○big    ○very big

## Supporting information

S1 File Interview outline file. (DOCX)

S2 File Expert information file. (XLSX)

S3 File Consent letter file. (DOCX)

S4 File Questionnaire original data file. (XLSX)
