# Supplementary material for: Factors affecting prefabricated construction promotion in China: A structural equation modeling approach
Source: PLoS One. 2020 Jan 27;15(1):e0227787. doi: 10.1371/journal.pone.0227787 (PMC6984738; doi:10.1371/journal.pone.0227787)
Supplement: S1 File — (DOCX) [file pone.0227787.s001.docx]

Dear Expert

In order to study the factors affecting prefabricated construction in China for your opinion. Please answer the following questions carefully:

1. What do you think should be emphasized in the design of the questionnaire?

2. Do you think the provisional list of factors is appropriate and which factor need to be deleted or added as shown in Table1?

3. Can you talk about your views on Chinese prefabricated construction industry?

4. What do you think should be paid attention to in the selection of the survey object in this study?

Table1 provisional factor list

| **Number** | **Observation variables** |
| --- | --- |
| 1 | Policy incentive |
| 2 | Industry standard |
| 3 | Regulatory mechanism |
| 4 | Policy guidance |
| 5 | BIM technology |
| 6 | Standardization |
| 7 | Technical talent |
| 8 | Integration |
| 9 | Organizational strategy |
| 10 | Information collaboration |
| 11 | Management mode |
| 12 | Industry chain |
| 13 | Public acceptance |
| 14 | Enterprise transformation |
| 15 | Production cost |
| 16 | Purchase cost |
| 17 | Transportation cost |
| 18 | Environmental performance |
| 19 | Economic performance |
| 20 | Social performance |

Thank you for taking the time to participate in this interview!

Research Group on Management Strategy and Coordination

Mechanism of Prefabricated Construction Supply Chain Driven by Big Data
