# Supplementary material for: Factors affecting prefabricated construction promotion in China: A structural equation modeling approach
Source: PLoS One. 2020 Jan 27;15(1):e0227787. doi: 10.1371/journal.pone.0227787 (PMC6984738; doi:10.1371/journal.pone.0227787)
Supplement: S3 File — (DOCX) [file pone.0227787.s003.docx]

Interview Consent

Dear Sir/Madam,

With the progress of urbanization in China, prefabricated construction is getting more and more attention in Chinese construction industry, but its promotion is slow. Our research group hopes to explore the reasons for its slow promotion by investigating the factors affecting the promotion of prefabricated construction.

The interview results and all the information obtained in the interview will only be used for the study of affecting factors of the promotion of prefabricated construction. All participants in the interview and interview data collation personnel will ensure that the information will not be leaked.

Thank you very much for your support!

Research Group on Management Strategy and Coordination

Mechanism of Prefabricated Construction Supply Chain Driven by Big Data
